# Comparison of Dako HercepTest and Ventana PATHWAY anti-HER2 (4B5) tests and their correlation with silver *in situ* hybridization in lung adenocarcinoma

**DOI:** 10.1515/med-2021-0366

**Published:** 2021-10-06

**Authors:** Mirjana Miladinović, Ljiljana Vučković, Aleksandra Klisic

**Affiliations:** Department of Pathology, Clinical Center of Montenegro, University of Montenegro-Faculty of Medicine, Podgorica, Montenegro; Center for Laboratory Diagnostics, Primary Health Care Center, University of Montenegro-Faculty of Medicine, Trg Nikole Kovacevica 6, 81000 Podgorica, Montenegro

**Keywords:** adenocarcinoma, amplification, HER2, IHC, lung

## Abstract

**Background:**

Discordant results exist about the role of human epidermal growth factor receptor 2 (HER2) overexpression and/or HER2 amplification in lung adenocarcinoma. We aimed to compare the performance of HercepTest and PATHWAY anti-HER2 (4B5) by correlating immunohistochemistry (IHC) results with silver *in situ* hybridization (SISH) in adenocarcinoma lung specimens.

**Methods:**

A total of 148 surgically resected adenocarcinoma lung specimens were included.

**Results:**

HER2 overexpression was found in 7.4% patients for HercepTest Dako and in 2.7% patients for 4B5 antibody. The overall coincidence between these two types of antibodies equals 93.9%. The incidence of HER2 amplification in lung adenocarcinoma was 17.6%, of which in 2.7% of the cases high-grade amplification was present. HER2 amplification was present in 90.9% of patients with overexpression of HER2, obtained by using HercepTest Dako and 75% patients using 4B5 antibody. A significant correlation between overexpression of HER2 receptors obtained by HercepTest Dako and 4B5 antibody and HER2 amplification was shown.

**Conclusion:**

The research of the efficiency of targeted molecular therapies with an HER2 antibody may serve as a basis for the introduction of routine HER2 status determination in lung adenocarcinoma, dictating the need for the standardized protocol for HER2 status determination in such pathology.

## 1 Introduction

Lung cancer is a heterogeneous disease and mutation profiling has now become a routine practice in pulmonary oncology [1]. The Lung Cancer Mutation Consortium was established in 2008 as a multinational programme investigating the frequency of selected oncogenic mutations in lung adenocarcinoma and using the obtained results for the application of targeted therapies. Oncogenes were detected in 64% of lung adenocarcinomas [2], while available information in relation to EGFR, BRAF, KRAS, ALK, ROS1, and MET gene mutations in lung adenocarcinomas are growing steadily. Among the proto-oncogene products, the epidermal growth factor (EGF) receptor family plays an important role in local tumour growth. Human epidermal growth factor receptor 2 (HER2) protein overexpression and/or HER2 gene amplification has a key role in the development and progressing of many carcinomas, especially breast and stomach cancer [3,4], but also in non-small cell lung carcinoma (NSCLC) [5,6]. HER2 belongs to the group of transmembrane receptors with tyrosine kinase activity, which modulate the transcription of genes involved in key cellular functions, including cell survival, proliferation, angiogenesis, ability of invasion, and metastasis [7]. The frequency of HER2 overexpression and HER2 gene amplification in NSCLC varies significantly from one study to another, but the majority of authors agree that overexpression and amplification are most prevalent in adenocarcinomas as compared to other histological types.

The prognostic significance of HER2 status determination in lung adenocarcinomas is controversial [8,9] and may vary depending on the applied detection method, immunohistochemistry (IHC), or *in situ* hybridization (ISH) [10].

Trastuzumab (HerceptinR), a human monoclonal antibody that recognizes the HER2 receptor, is in the research phase for the treatment of HER2-positive NSCLC. Transtuzumab is approved in the treatment of breast cancer and gastric cancer in cases of HER2 overexpression or amplification. This drug is not yet an integral part of standard NSCLC treatment, but according to the results of Zhao et al. certain patients may benefit from this therapy [11]. In the studies conducted so far, the greatest potential benefit of targeted therapies in patients with NSCLC has been observed in the group of patients with an HER2 overexpression, which is most common in poorly differentiated adenocarcinomas [12,13]. The research of the efficiency of targeted molecular therapies, with an HER2-specific human monoclonal antibody, may serve as a basis for the introduction of routine HER2 status determination in lung adenocarcinoma, which dictates the need for the standardized protocol for HER2 status determination in lung adenocarcinoma. Currently, HER2 IHC and ISH diagnostic tests are used to determine the HER2 status. The results may vary depending on the type of antibodies used, antibody concentration, tissue preservation, as well as subjective bias when reading the results.

The goal of our study was to compare the performance of HercepTest and PATHWAY anti-HER2 (4B5) by correlating IHC results with silver *in situ* hybridization (SISH) in adenocarcinoma lung specimens.

## 2 Materials and methods

### 2.1 Patients selection

The study was approved by the Ethics Committee of the Institute for Pulmonary Diseases of Vojvodina (IPDV), (number 77-V/24, 30 May 2017) an informed consent was obtained from each patient. The samples were obtained from 148 patients who had surgical resection of primary lung adenocarcinoma at the IPDV (Serbia), from January 2010 to December 2017. Clinical pathological information were collected for each patient, including age, sex, smoking history, maximum tumour size (in cm), and pathological stage (p-stage). All tumour tissue samples were classified in accordance with the International Association for the Study of Lung Cancer/American Thoracic Society/European Respiratory Society (IASLC/ATS/ERS) for adenocarcinoma [14]. The 8th edition of the TNM stage classification for lung cancer was used for pathological TNM classification and staging [15].

### 2.2 Tissue microarray (TMA)

After histopathological evaluation of the specimens of all 148 patients, paraffin-embedded tumour blocks with sufficient tissue were selected for preparation of a TMA. The most representative region of the tumour was selected based on the morphology of the H&E-stained slide. Tissue cores were punched out from each donor tumour block using thin-walled 3 mm stainless steel needles (Quick-Ray Manual Tissue Microarrayer), and cores were arrayed in a recipient paraffin block. All subsequent tests (IHC and ISH) were performed on serially cut 4 μm paraffin-embedded tissue sections.

### 2.3 HercepTest (Dako)

From 5 TMA molds where the tissue samples of all 148 patients were inserted, cuts 4 microns wide were sliced and caught onto Superfrost glass slides, and then dried for 30 min 60°C. After that, dying was performed with Dako’s primary human, rabbit’s HER2 antibody, by applying LSAB+/HRP visualization method. After deparrafinization in xylene and rehydration through series of alcohols of decreasing concentration, samples of all 148 patients were dyed with application of HercepTest Dako, respecting the following protocol for manual conduction.

### 2.4 PATHWAY anti-HER2 (4B5) (Ventana)

Staining was performed on BenchMark®XT automated slide stainer (I-VIEV put HER2/neu kit, Ventana Medical Systems) following the standard preprogramed staining protocol. Briefly, an automated deparaffinization step was followed by cell conditioning (antigen unmasking) for 38 m and then rinsed with a reaction buffer and incubated with the prediluted anti-HER2 rabbit monoclonal primary antibody (clone 4B5) at 37°C for 16 min. After rinsing with the reaction buffer, staining was visualized using the ultraView Universal DAB Detection Kit.

### 2.5 IHC Scoring system

Immunohistochemical staining was performed with quality control and dying specificities with application of external and internal control. Well-known breast carcinoma tissue samples proven for excessive protein expression of HER2 graded with 3+ were used as positive control samples. For interpretation of IHC expression of HER2, a modified pattern of the HercepTest for determining protein expression of HER2 in breast carcinoma was used [9]: tumours with complete absence of dying – 0; tumours with poor, incomplete membrane – 1+; tumours with strong, incomplete basolateral dying or poor, complete membrane dying in more than 10% of tumour cells – 2+; tumours with strong, complete membrane dying in more than 10% of tumour cells – 3+. The tumour tissue samples that were graded with 2+ or 3+ were regarded as positive, while the tumour tissue samples graded with 0 and 1+ were regarded as negative.

### 2.6 Silver *in situ* hybridizatin and interpretatin

SISH was performed using the INFORM HER2 Dual ISH DNA Probe Cocktail assay (Ventana Medical Systems) with an automated slide stainer according to the manufacturer’s protocols (BenchMark XT; Ventana Medical Systems). HER2 gene status was determined on the basis of counting its copies (black signals) and detecting the chromosome 17 centromere (red signals) in at least 20 tumour cells. Amplification of the HER2 gene was determined according to the ASCO/CAP guidelines for dual-probe ISH [16].

### 2.7 Statistical analysis

Statistical data processing was done with statistics program SPSS 20.0 (SPSS Inc, Chicago, IL, USA). The obtained results were described with standard statistical variables (medium value (*n*), standard deviation (SD), and interval values (max and min)). Chi-square test and Fisher test were used for testing the difference between examined variables. For all statistical analyses, *P* value <0.05 was regarded as statistically significant.

## 3 Results

### 3.1 Patients’ characteristics

Among the 148 patients who underwent invasive lung adenocarcinoma surgery for the first time at the IPDV, 62.2% were male and 37.8% were female. The average age of the total sample was 60.8 ± 7.87. As regards their smoking habits, 5.4% were non-smokers, 18.9% were ex-smokers, while 75.7% were active smokers. The average tumour size amounted to 48.2 ± 26.4 mm. Table 1 shows the characteristics of patients with lung adenocarcinoma. The following histological types of adenocarcinoma were included in the study: solid (*n* = 52), acinar (*n* = 46), papillary (*n* = 25), micropapillary (*n* = 15), lepidic (*n* = 8), and enteric (*n* = 2).

**Table 1:** Patients’ characteristics according to HER2 protein overexpression

	Total	HER2 protein expression HercepTest (Dako)			HER2 protein expression PATHWAY (45B)		
		Positive	Negative	*P* value	Positive	Negative	*P* value
Characteristics, *n* (%)	148 (100)	11 (7.4)	137 (92.6)		4 (2.7)	144 (97.3)	
Gender							*P* = 1
Male	92 (62.2)	8 (5.4)	84 (56.8)		3 (2)	89 (60.1)	
Female	56 (37.8)	3 (2)	53 (35.8)		1 (0.7)	55 (37.2)	
Median age, 60.8 ± 7.87 (range: 32–79)							
Age (years)				*P* = 0.562			*P* = 0.611
31–40	1 (0.7)	0 (0)	1 (0.7)		0 (0)	1 (0.7)	
41–50	13 (8.8)	0 (0)	13 (8.8)		0 (0)	13 (8.8)	
51–60	57 (38.5)	4 (2.7)	53 (35.8)		1 (0.7)	56 (37.8)	
61–70	64 (43.2)	5 (3.4)	59 (39.9)		2 (1.4)	62 (41.9)	
71–80	13 (8.8)	2 (1.4)	11 (7.4)		1 (0.7)	12 (8.1)	
Smoking status				*P* = 0.713			*P* = 0.1
Current	112 (75.7)	8 (5.4)	104 (70.3)		3 (2)	109 (73.6)	
Never	8 (5.4)	0 (0)	8 (5.4)		0 (0)	8 (5.4)	
Former	28 (18.9)	3 (2)	25 (16.9)		1 (0.7)	27 (18.2)	
Tumour size, mm (range), 48.2 ± 26.4 (range: 9–150)				*P* = 0.700			*P* = 0.891
0–20	15 (10.1)	0 (0)	15 (10.1)		0 (0)	15 (10.1)	
21–30	24 (16.2)	1 (0.7)	23 (15.5)		1 (0.7)	23 (15.5)	
31–50	59 (39.9)	6 (4.1)	53 (35.8)		2 (1.4)	57 (38.5)	
>50	50 (33.8)	4 (2.7)	46 (31.1)		1 (0.7)	49 (33.1)	
T status				*P* = 0.993			*P* = 1
T1a	14 (9.5)	1 (0.7)	13 (8.8)		0 (0)	14 (9.5)	
T1b	22 (14.9)	1 (0.7)	21 (14.2)		1 (0.7)	21 (14.2)	
T2a	46 (31)	4 (2.7)	42 (28.4)		1 (0.7)	45 (30.4)	
T2b	24 (16.2)	2 (1.4)	22 (14.9)		1 (0.7)	23 (15.5)	
T3	37 (25)	3 (2.0)	34 (23.0)		1 (0.7)	36 (24.3)	
T4	5 (3.4)	1 (0.7)	4 (2.7)		0 (0)	5 (3.4)	
N status				*P* = 0.584			*P* = 0.596
N0	96 (64.9)	9 (6.1)	87 (58.8)		3 (2)	93 (62.8)	
N1	31 (20.9)	2 (1.4)	29 (19.6)		0 (0)	31 (20.9)	
N2	19 (12.8)	0 (0)	19 (12.8)		1 (0.7)	18 (12.2)	
N3	2 (1.4)	0 (0)	2 (1.4)		0 (0)	2 (1.4)	
M status				*P* = 0.499			*P* = 1.000
M0	124 (83.8)	11 (7.4)	113 (76.4)		4 (2.7)	120 (81.1)	
M1a	3 (2)	0 (0)	3 (2)		0 (0)	3 (2)	
M1b	21 (14.2)	0 (0)	21 (14.2)		0 (0)	21 (14.2)	
Pathologic stage				*P* = 0.543			*P* = 0.861
IA	22 (14.9)	2 (1.4)	20 (13.5)		1 (0.7)	21 (14.2)	
IB	28 (18.9)	4 (2.7)	24 (16.2)		1 (0.7)	27 (18.2)	
IIA	24 (16.2)	2 (1.4)	22 (14.9)		0 (0)	24 (16.2)	
IIB	26 (17.6)	1 (0.7)	25 (16.9)		1 (0.7)	25 (16.9)	
IIIA	22 (14.9)	2 (1.4)	20 (13.5)		1 (0.7)	21 (14.2)	
IIIB	2 (1.4)	0 (0)	2 (1.4)		0 (0)	2 (1.4)	
IV	24 (16.2)	0 (0)	24 (16.2)		0 (0)	24 (16.2)	

HER2, human epidermal growth factor receptor 2; *n*, number; T, tumour; N, node; M, metastasis; *P* value < 0.05, non-parametric Fisher’s test.

### 3.2 HER2 IHC

**Figure 1:**
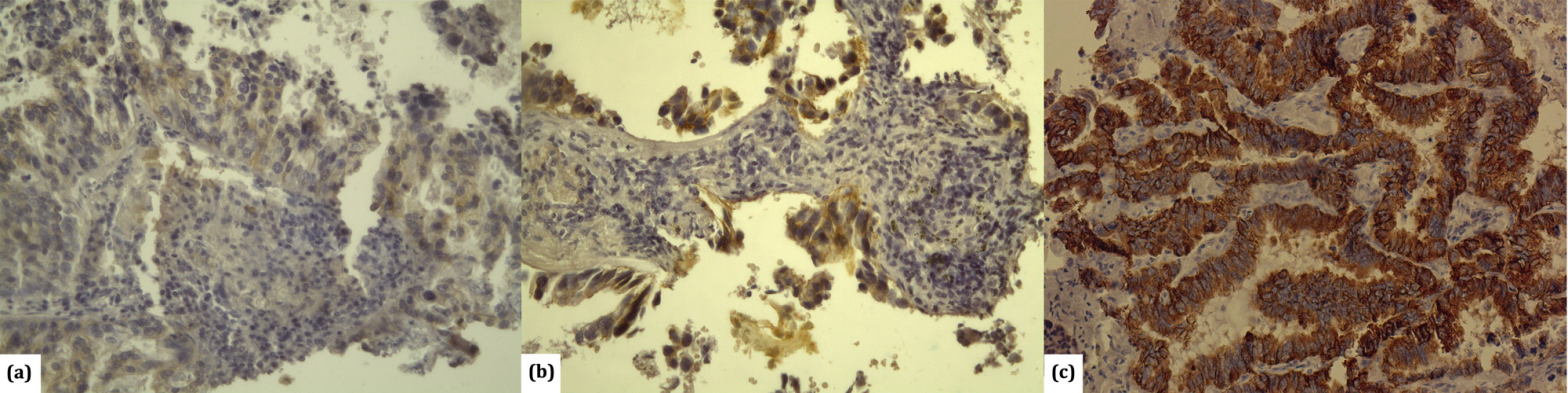
Immunohistochemical detection of HER-2 antibody HercepTest, Dako in lung adenocarcinoma: (a) tumours with poor, incomplete membrane – negative 1+ (×200); (b) tumours with strong, incomplete basolateral dying in more than 10% of tumour cells – positive 2+ (×200); and (c) tumours with strong, complete membrane dying in more than 10% of tumour cells-positive 3+ (×200).

**Figure 2:**
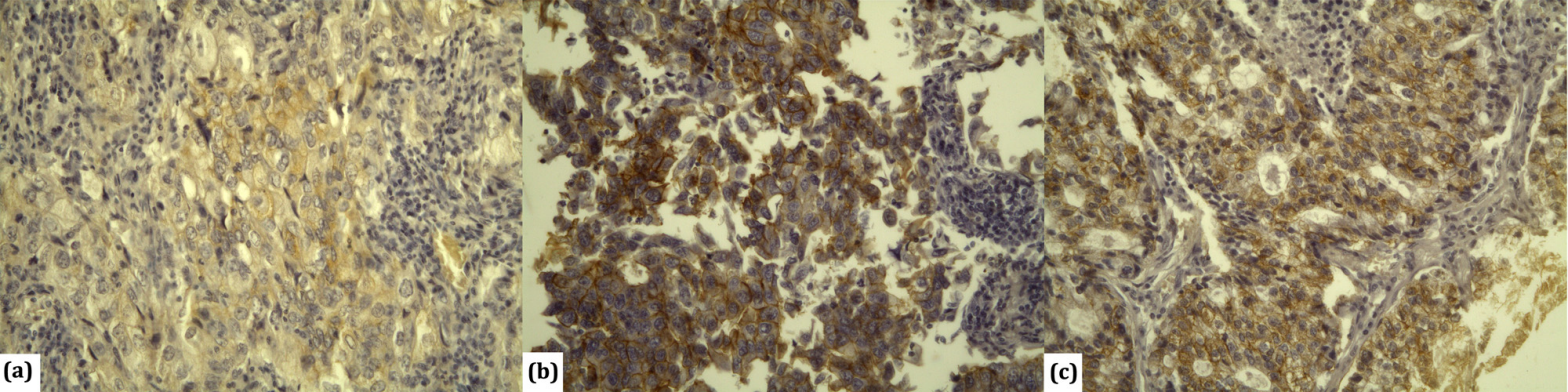
Immunohistochemical detection of HER2 antibody Ventana anti-HER2/neu (4B5) in lung adenocarcinoma: (a) tumours with poor, incomplete membrane – negative 1+ (×200); (b) tumours with strong, incomplete basolateral dying in more than 10% of tumour cells – positive 2+ (×200); and (c) tumours with strong, complete membrane dying in more than 10% of tumour cells-positive 3+ (×200).

By applying HercepTest (Dako) antibodies, HER2 overexpression in patients with lung adenocarcinoma was detected in 11 (7.4%) patients, among whom the score was 2+ for 6 (4%) patients and 3+ for 5 (3.4%) patients (Figure 1). By applying PATHWAY anti-HER2 (4B5) antibodies, overexpression was detected in 4 (2.7%) patients, among whom the score was 2+ for 3 (2%) patients and 3+ for 1 (0.7%) patient (Figure 2).

By testing the coincidence between the results of HER2 overexpression obtained by applying HercepTest (Dako) antibodies and the results obtained by applying PATHWAY anti-HER2 (4B5) antibodies, coincidence was established in three cases (2% of the total samples). When it comes to negative HER2 protein expression, the results obtained by applying these two types of antibodies were identical in 136 cases (91.9%) (Table 2).

**Table 2:** Comparison of Hercep test (Dako) and PATHWAY (4B5-Ventana) IHC results

HercepTest *N* (%)			
PATHWAY (45B)	Negative (0–1+)	Positive (2+)	Positive (3+)
Negative (0–1+)	136 (91.9)	4 (2.7)	4 (2.7)
Positive (2+)	1 (0.7)	1 (0.7)	1 (0.7)
Positive (3+)	0 (0)	1 (0.7)	0 (0)
Total (*n* = 148)	137	6	5

*N*, number.

By using the non-parametric Fisher’s test, the obtained *P* value (*P* = 0.001) indicates a high statistical correlation between the results obtained by applying these two types of antibodies. The overall coincidence between these two types of antibodies equals 93.9%. The correlation coefficient Phi amounted to 0.429 for nominal values, which proved a medium-high correlation between the results obtained by applying the HercepTest Dako and Ventana anti-HER2/neu (4B5) antibodies.

### 3.3 HER2 gene amplification

**Figure 3:**
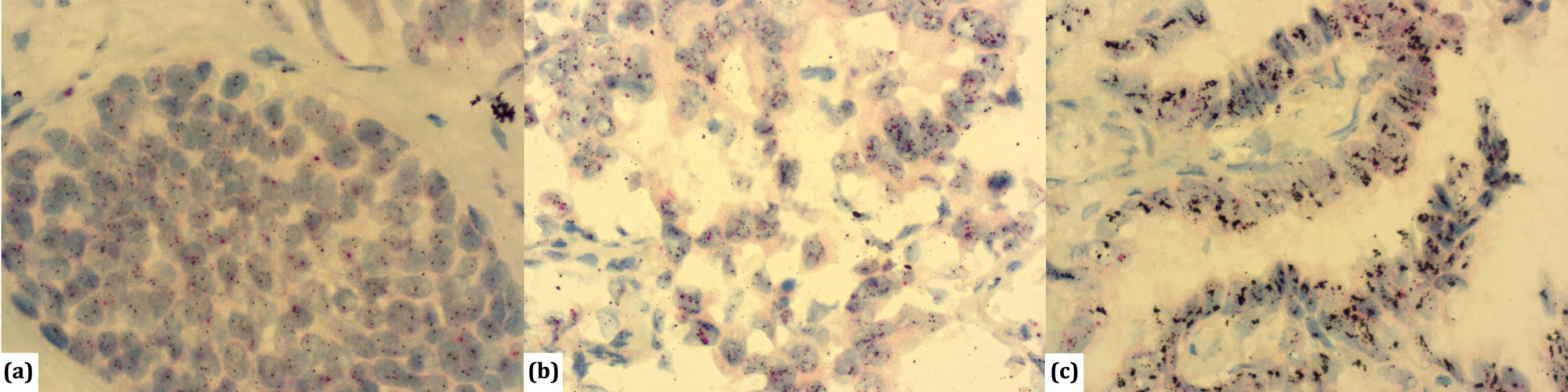
SISH method for detection of *HER2* gene (black label) and *CEP17* (red label) in lung adenocarcinoma: (a) *HER2*/*CEP17* ratio *<*2.0 with an average *HER2* copy number <4.0 signals/cell-absence of HER2 amplification, (×600); (b) *HER2*/*CEP17* ratio ≥2.0 with an average *HER2* copy number ≥4.0 signals per cell – positive amplification HER2 gene (×600); (c) tumours harboring an average copy number of ≥10.0 signals/cell-*high-level* amplification HER2 gene (×600).

The presence of HER2 amplification through ISH (Dual IHC HER2 kit; Ventana Medical Systems) was tested in all 148 patients. Amplification was present in 26 patients, among whom *high-grade* amplification was found in four patients (Figure 3). The frequency of HER2 amplification in adenocarcinoma was 17.6% of the total sample, out of which 2.7% was *high-grade* amplification. The distribution of HER2 overexpression and gene amplification in relation to the predominant histological type of lung adenocarcinoma is shown in Table 3.

**Table 3:** Distribution of HER2 expression and amplifications in the predominant adenocarcinoma histology

Predominant type	Total	HER2 expression HercepTest (Dako) *N* (%)	HER2 expression PATHWAY (45B) *N* (%)	HER2 amplification *N* (%)
Solid	52 (35.1)	5 (45.4)	1 (25)	11 (42.3)
Acinar	46 (31.1)	4 (36.4)	3 (75)	7 (26.9)
Papillary	25 (16.9)	1 (9.1)	0 (0)	6 (23.1)
Micropapillary	15 (10.1)	1 (9.1)	0 (0)	2 (7.7)
Lepidic	8 (5.4)	0 (0)	0 (0)	0
Enteric	2 (1.4)	0 (0)	0 (0)	0
Total	148	11	4	26

HER2, human epidermal growth factor receptor 2; *N*, number.

### 3.4 Correlation of IHC with ISH

The presence of HER2 amplification and HER2 overexpression obtained through HercepTest Dako was found in 10 patients (6.8% of the total sample). On the other hand, the absence of HER2 amplification was found in 121 (81.8%) patients who did not have an HER2 protein overexpression. The application of the non-parametric Fisher’s test established a statistically significant correlation between the amplification determined by ISH and the HER2 receptor expression obtained through HercepTest Dako (*P* < 0.001). The Phi coefficient value indicates a high correlation of the results obtained through these two tests (Phi = 0.546).

The presence of HER2 amplification and HER2 overexpression obtained through Ventana anti-HER2/neu (4B5) was found in three patients (2% of the total sample). The absence of HER2 gene amplification was found in 121 patients (81.8% of the total sample). The non-parametric Fisher’s test indicated a significant correlation between the amplification determined by ISH and the HER2 expression obtained through Ventana anti-HER2/neu (4B5) (*P* = 0.017). The Phi coefficient value indicates a low correlation of the results obtained through these two tests (Phi = 0.252) (Table 4).

**Table 4:** Correlation of SISH and IHC results

IHC *N* (%)						
	HercepTest			PATHWAY (4B5)		
SISH	0/1+	2+	3+	0/1+	2+	3+
No amplification	121 (81.8)	1 (0.7)	0 (0)	121 (81.8)	1 (0.7)	0 (0)
Amplification	16 (10.8)	5 (3.4)	5 (3.4)	23 (15.5)	2 (1.3)	1 (0.7)
Total (*n* = 148)	137	6	5	144	3	1
*P* value	***P*** < **0.001**			***P*** = **0.017**		
Phi	0.546			0.252		

SISH, silver *in situ* hybridization; IHC, immunohistochemistry; *N*, number; *P* value < 0.05, non-parametric Fisher’s test; Phi correlation coefficient.

## 4 Discussion

To our knowledge, our study is the first study to use the same samples of lung adenocarcinoma tumour tissue for the IHC determination of HER2 expression by applying two different antibodies (Hercep Test Dako and Ventana anti-HER2/neu (4B5)).

The therapeutic effect of targeted HER2 monoclonal antibodies that have been used for NSCLC so far (neratinib, dacomitinib, lapatinib, and afatinib) is not completely clear [17,18]. However, a correct evaluation of the HER2 status plays an important role in the assessment of this therapeutic effect. Correct evaluation tests and well-defined detailed criteria are necessary for an appropriate selection of patients that might benefit from a targeted therapy.

The studies that assessed HER2 expression in NSCLC by applying IHC provided different data. In our research, an IHC determination of HER2 expression was performed for all patients using two antibodies: Hercep Test Dako and Ventana anti-HER2/neu (4B5), and the obtained results were then compared. By analysing the frequencies of positive and negative HER2 expression, we established that positive HER2 overexpression (2+, 3+) in patients with lung adenocarcinoma amounts to 7.4% for HercepTest Dako, and 2.7% for Ventana anti-HER2/neu (4B5). In the literature, the prevalence of protein overexpression ranges between 2.4 and 38% [5,19,20,21]. By applying HercepTest Dako, other authors obtained a percentage-wise higher prevalence of HER2 overexpression in lung adenocarcinoma in comparison to our results: Awaya et al. 20.9% [22], Yoshizawa et al. 15.2% [20], and Grob et al. 13.9% [23]. When the Ventana anti-HER2/neu (4B5) antibody is used for determining HER2 overexpression, our results are similar to the results of other studies. Suzuki et al. published an HER2 positivity of 2.6% on a sample of 1055 adenocarcinomas [6], while a slightly more frequent HER2 overexpression was reported by Kobyakov et al. –6.1% [24] and Kim et al. –7.8% [25].

In relation to the histological type of adenocarcinoma, HER2 overexpression obtained through HercepTest Dako was more frequent in solid (45.5%) and acinar (36.4%), than in papillary (9.1%) and micropapillary (9.1%) adenocarcinomas. In lepidic and enteric cases of adenocarcinoma, HER2 receptor overexpression was not detected. Kim et al. obtained the highest HER2 receptor protein overexpression in papillary-predominant adenocarcinoma (50%), the second most frequent was acinar (33.3%), the third was mucinous (16.7%), while other types showed no overexpression [25]. Li et al. reported the highest prevalence of overexpression in the acinar type (38.5%), followed by the papillary (23.1%) and the solid (15.4%) types, while other types were represented by low percentages [26]. When comparing our data with the data of other authors, we can conclude that the prevalence of positive HER2 receptor expression obtained through HercepTest Dako is similar when it comes to the acinar type of adenocarcinoma. As opposed to other authors, where the papillary type of adenocarcinoma was often accompanied by HER2 overexpression, in our study HER2 overexpression was most often found in the solid type of adenocarcinoma. When it comes to micropapillary, lepidic, and enteric types, our data do not deviate from the results of other authors, i.e. these types are rarely accompanied by HER2 protein overexpression.

The presence of HER2 receptor protein overexpression obtained through Ventana anti-HER2/neu (4B5) was found in the acinar (75%) and solid (25%) types of adenocarcinoma. In a study on a sample of 321 adenocarcinomas, Kim et al. found HER2 receptor protein expression most often in papillary carcinomas (50%), which differs from our results where papillary-predominant tumours did not show HER2 overexpression. In the same study, the acinar type was represented merely by 33.3% (the second most common histological type), which is a significantly lower percentage than that in our study [25]. Li et al. [26] reported the most frequent positive HER2 overexpression in the acinar-predominant type (38.5%), which is a lower percentage, but it still represents the most prevalent histological type as in our study. They reported the papillary-predominant type as the second most frequent (23.1%). In our study, we did not find HER2 overexpression in papillary type cases. According to their study, the solid type was accompanied by HER2 receptor overexpression in 15.4% of the cases, which is similar to our results [26]. Suzuki et al. reported that there was no correlation between HER2 overexpression (obtained by applying Ventana anti-HER2/neu (4B5) antibodies) and the predominant histological type of adenocarcinoma [6].

In our research by comparing the results of (positive) HER2 overexpression obtained through the use of HercepTest Dako antibodies and the results obtained through the use of Ventana anti-HER2/neu (4B5) antibodies, an overlap was established in three cases, i.e. 2% of the total sample. When it comes to negative HER2 protein expression, these two antibodies overlapped in 136 cases (91.9%). Larger number of patients (12) showed HER2 overexpression after the use of Hercep Test Dako, in comparison to only four patients in whom protein overexpression was detected after the use of Ventana anti-HER2/neu (4B5) antibodies. By analysing the percentage-wise prevalence of HER2 overexpression in lung adenocarcinoma in studies that used HercepTest Dako and studies that used Ventana anti-HER2/neu (4B5) antibodies, we observed a higher prevalence when using Hercep Test Dako as compared to Ventana anti-HER2/neu (4B5) antibodies, which is also the case in our study [6,20,22,23,24,25]. By using the non-parametric Fisher’s test, the obtained value *P* = 0.001 indicates a high statistical correlation between the results obtained by applying these two types of antibodies. The overall coincidence between these two types of antibodies equals 93.9%. The correlation coefficient Phi amounted to 0.429 for nominal values, which proved a medium-high correlation between the HercepTest Dako and Ventana anti-HER2/neu (4B5) antibodies.

The presence of HER2 amplification through ISH (Dual IHC HER2 kit; Ventana Medical Systems) was tested in all 148 patients. Amplification was present in 26 patients (17.6%), among whom high-grade amplification was found in four patients (2.7%). According to the published data, the frequency of HER2 amplification ranges between 10 and 20% in NSCLC [27,28,29,30], whereas high-grade amplification is rare and accounts for only 1–5% [29,30]. Kim et al. reported an amplification frequency of 14.3% on a sample of 321 adenocarcinomas, with a high-grade amplification of 1.6% [25]. Kobyakov et al. recorded an HER2 gene amplification in 16.6% of adenocarcinomas, with a high-grade amplification of 2% [24]. The amplification results on our sample correspond to the data published by other authors.

On our sample, the presence of HER2 gene amplification was more frequent in solid (42.3%), acinar (27%), and papillary (23%) types as compared to the micropapillary type (7.7%). In the group of lepidic and enteric types of adenocarcinoma, no amplification was found. Kim et al. recorded HER2 gene amplification most frequently in predominantly acinar adenocarcinomas (58.7%), followed by papillary (15.2%), micropapillary (13%), and solid (8.7%), while amplification was the least frequent in lepidic adenocarcinomas (4.3%) [25]. Unlike other authors, in our study the solid type of adenocarcinoma was most frequently accompanied by HER2 amplification.

Many studies have shown the compliance between HER2 IHC and ISH in breast carcinoma, but only a small number of studies have demonstrated such compliance in NSCLC [31].

By analysing HER2 gene amplification in samples where HER2 overexpression was obtained through the use of HercepTest Dako, we observed the following connection. The presence of HER2 gene amplification coincides with HER2 overexpression obtained through HercepTest Dako in 10 (90.9%) of a total of 11 patients with HER2 overexpression. On the other hand, a negative status of HER2 gene amplification corresponds with the absence of HER2 overexpression obtained through Hercep Test Dako in 121 (81.8%) patients out of the total sample. The application of the non-parametric Fisher’s test established a correlation between HER2 gene amplification determined by “Dual ISH HER2 kit; Ventana Medical Systems” and the HER2 receptor expression obtained through HercepTest Dako (*P* < 0.001). The Phi coefficient value indicates a high correlation of the results obtained through these two tests (Phi = 0.546). Other authors have also reported a significant correlation between HER2 gene amplification and HER2 expression obtained through the use of HercepTest Dako [20,23]. On a sample of 243 lung adenocarcinomas and using Hercep Test Dako, Yoshizawa et al. detected HER2 overexpression in 37 patients (score 2+ and 3+), among whom HER2 gene amplification was found in four patients using “Dual ISH, Ventana Medical Systems” [20]. Our sample shows a significantly higher coincidence between HER2 overexpression (HercepTest Dako) and gene amplification (10/11), as compared to other studies (4/37) [20], 2/16 [22].

By comparing the presence of HER2 amplification and HER2 overexpression obtained through Ventana anti-HER2/neu (4B5) in our sample, positive findings were observed in three out of four patients (75%). The absence of HER2 amplification was found in 81.8% of the patients out of the total sample who did not have HER2 protein overexpression. We have also found a significant correlation between HER2 gene amplification and HER2 receptor protein expression obtained through Ventana anti-HER2/neu (4B5) antibodies. The correlation between amplification and HER2 expression obtained through Ventana anti-HER2/neu (4B5) antibodies has been reported by other authors as well [6,20,25], while the results of some authors negated such correlation [30]. In a study that tested the clinical significance of HER2 alterations in lung adenocarcinoma, Kim et al. used the same methodology to determine the amplification and HER2 receptor expression (Dual ISH, Ventana Medical Systems; Ventana anti-HER2/neu (4B5)). Out of 321 patients with lung adenocarcinoma, they found HER2 overexpression in 25 patients, while gene amplification was present in 11 of those patients (44%). They detected an absence of HER2 gene amplification and HER2 overexpression in 81.3% of patients out of the total sample. They proved a statistically significant correlation between HER2 receptor protein expression and gene amplification, albeit with a low degree of correlation. Our sample shows a quite greater overlap between protein overexpression and gene amplification (3/4) in comparison to the results of other studies (11/25). Both in our study and in the results of other authors, HER2 gene amplification is detected more often as compared to HER2 overexpression [25].

This study has certain limitations. First, this study is retrospective, conducted in a single institution. Second, the size of our sample is relatively small. Thus, a larger sample in such a study could yield different results. Third, the accuracy of the results could be affected by any possible bias when reading the results. In order to reduce any bias to the lowest possible degree, each sample was assessed independently by two pathologists. The final result was the product of a consensus. In cases of disagreements between the two pathologists, the opinion of a third pathologist – whose daily work dominantly includes lung pathology and who has the most years of work in this field – was sought.

In conclusion, HER2 protein overexpression in lung adenocarcinoma amounts to 7.4% for Hercep Test Dako and 2.7% for Ventana anti-HER2/neu (4B5) antibodies. For positive expression and negative expression, the results of these two tests correspond in 2 and 91.9% of the cases, respectively, which amounts to a total of 93.9%. The frequency of HER2 gene amplification in lung adenocarcinoma is 17.6%, out of which 2.7% of the cases include *high-grade* amplification. HER2 gene amplification is present in 90.9% of the patients with HER2 receptor protein overexpression, which is obtained by using HercepTest Dako, and in 75% when using Ventana anti-HER2/neu (4B5) antibodies.
